# Toward inclusive primary health care: understanding health needs of women in India’s informal economy through a socioecological framework

**DOI:** 10.1186/s12913-025-13855-7

**Published:** 2025-12-06

**Authors:** Ankit Sheth, Rakesh Balachandar, Ankit Viramgami, Anuj Dave, Ekta Ram, Zulekha Khalil, Mahendra Thakor

**Affiliations:** 1 ICMR-National Institute of Occupational Health, Ahmedabad, 380016 India; 2 ICMR – Regional Occupational Health Centre (South), Bengaluru, 562110 India; 3 Self-Employed Women’s Association (SEWA), Ahmedabad, India

**Keywords:** Informal sector, Musculoskeletal diseases, Occupational health, Female, Focus groups, Health policy, Socioecological model

## Abstract

**Background:**

Women in India’s informal economy face significant occupational health risks that remain largely undocumented and unaddressed. With limited labour protections and inadequate access to health services, informal women workers (IWWs) experience overlapping vulnerabilities related to gender, work conditions, and environmental exposures. This study explored the multi-level determinants of health among IWWs in Ahmedabad, India, to inform gender-responsive integration of occupational health within primary health care systems.

**Methods:**

A qualitative study was conducted using focus group discussions (FGDs) guided by the Socioecological Model (SEM). Five FGDs were held with 41 women representing key occupational groups—agricultural workers, construction workers, street vendors, home-based workers, and waste recyclers. Discussions were recorded, transcribed, translated, and thematically analysed. Themes were organized across SEM domains: intrapersonal, interpersonal, organizational, community, and policy levels.

**Findings:**

Participants reported multiple, intersecting health risks such as musculoskeletal disorders, respiratory problems, skin irritation, and heat-related illnesses. Psychological stress, economic insecurity, and work-family conflict were pervasive, compounded by gendered expectations and absence of social protection. Poor workplace infrastructure, including lack of sanitation and shade exacerbated illness and fatigue. Many women avoided drinking water due to lack of toilets, leading to dehydration and urinary problems. Health-seeking behaviour was shaped by trust and convenience; private clinics were preferred over public facilities despite higher costs. Awareness of government schemes such as Ayushman Bharat and e-Shram was limited. Participants expressed demand for pensions, maternity protection, and home-based livelihood support.

**Conclusion:**

Findings underscore the urgent need for gender-responsive occupational health integration into primary health care system. The study informed a national policy roundtable that convened key stakeholders to co-develop actionable recommendations to improve occupational health coverage for women in India’s informal economy.

**Supplementary Information:**

The online version contains supplementary material available at 10.1186/s12913-025-13855-7.

## Research in context

### Evidence before this study

We conducted a systematic review to identify published literature on the occupational health challenges faced by informal women workers (IWWs) in India. We searched PubMed, Scopus, and Embase databases without language restrictions for studies published between January 1, 2000, and December 31, 2023. Search terms included combinations of MeSH and free-text terms such as “informal women workers”, “occupational health”, “India”, “health challenges”, and “women in informal sector”. Studies were included if they assessed physical, mental, or occupational health issues among IWWs, excluding commentary articles and predictive modelling studies. Risk of bias was assessed using relevant tools based on study type. Due to heterogeneity in study designs and lack of uniform measurement tools, meta-analysis could not be conducted. However, descriptive synthesis revealed high burden of musculoskeletal disorders, mental stress, and reproductive health concerns, with limited access to social protection and healthcare.

### Added value of this study

This is one of the first studies to integrate qualitative data with a socioecological framework to explore multi-level health determinants for IWWs across five occupational groups in urban India. The study adds value by highlighting not only occupational exposures but also systemic, gendered, and policy-level barriers that impact access to healthcare and social safety nets. The inclusion of lived experiences and worker-driven recommendations enhances the contextual relevance and applicability of findings.

### Implications of all the available evidence

Combined with existing evidence, our findings reinforce the urgent need to integrate occupational health services within primary healthcare systems, particularly targeting women in India’s informal economy. Structural inequities, such as lack of identification mechanisms, poor sanitation infrastructure, and exclusion from social protection must be addressed to improve access and outcomes. This study provides actionable insights for policymakers, including recommendations on gender-sensitive occupational health integration, data collection at the primary health centre level, and capacity building of frontline healthcare providers.

## Introduction

Occupational health is a critical yet overlooked dimension of public health, particularly for informal sector workers who face hazardous environments without adequate protections. Informal employment—untethered from labour laws, social security provisions, and formal contracts—excludes workers from even basic occupational health and safety (OHS) standards [1]. In India, nearly 93% of the workforce is informal, with over 95% of women engaged in such work, reflecting pronounced gender-based vulnerabilities [2]. These women form the backbone of agriculture, construction, home-based manufacturing, waste recycling, and street vending, yet their occupational health remains poorly documented and insufficiently addressed.

Women in India’s informal economy (hereafter referred to as informal women workers, IWWs) face multiple health risks, including musculoskeletal disorders (MSD), respiratory illnesses, reproductive complications, skin infections, and mental health problems, driven by poor ergonomics, dust and chemical exposure, extreme temperatures, and inadequate infrastructure [3–6]. Gender further compounds these risks through wage disparities, job insecurity, and limited access to public services [7]. Poor nutritional status is also common, with implications for child health [8].

Environmental exposures add further strain. Outdoor workers are increasingly vulnerable to heat-related illnesses, dehydration, and injuries due to rising temperatures, air pollution, and water scarcity [9–11]. Despite growing evidence, occupational health policy and research in India remain largely focused on the formal workforce, leaving IWWs underrepresented in health planning and service delivery.

Some studies have documented sector-specific hazards, but few have used a multi-level framework to examine the broader social, environmental, and policy determinants that shape IWWs’ health. This limits understanding of how structural inequities – such as the absence of contracts, legal entitlements, and employment benefits – intersect with occupational exposures to restrict healthcare access, social protection, and policy inclusion. Moreover, the lived experiences of IWWs are rarely integrated into occupational health discourse or system planning, despite their importance for designing contextually appropriate, gender-responsive interventions.

This study addresses these gaps by applying the Socioecological Model (SEM) to examine the multi-layered health challenges of IWWs in Ahmedabad, Gujarat—a rapidly industrializing region with socioeconomic diversity and a dense informal labour force. Through focus group discussions (FGDs) across key occupational groups, it seeks to generate evidence grounded in workers’ experiences to inform strategies for integrating gender-responsive occupational health services within existing primary health care structures, thereby advancing equity and access for this underserved workforce.

## Methods

### Study design and setting

**Fig. 1:**
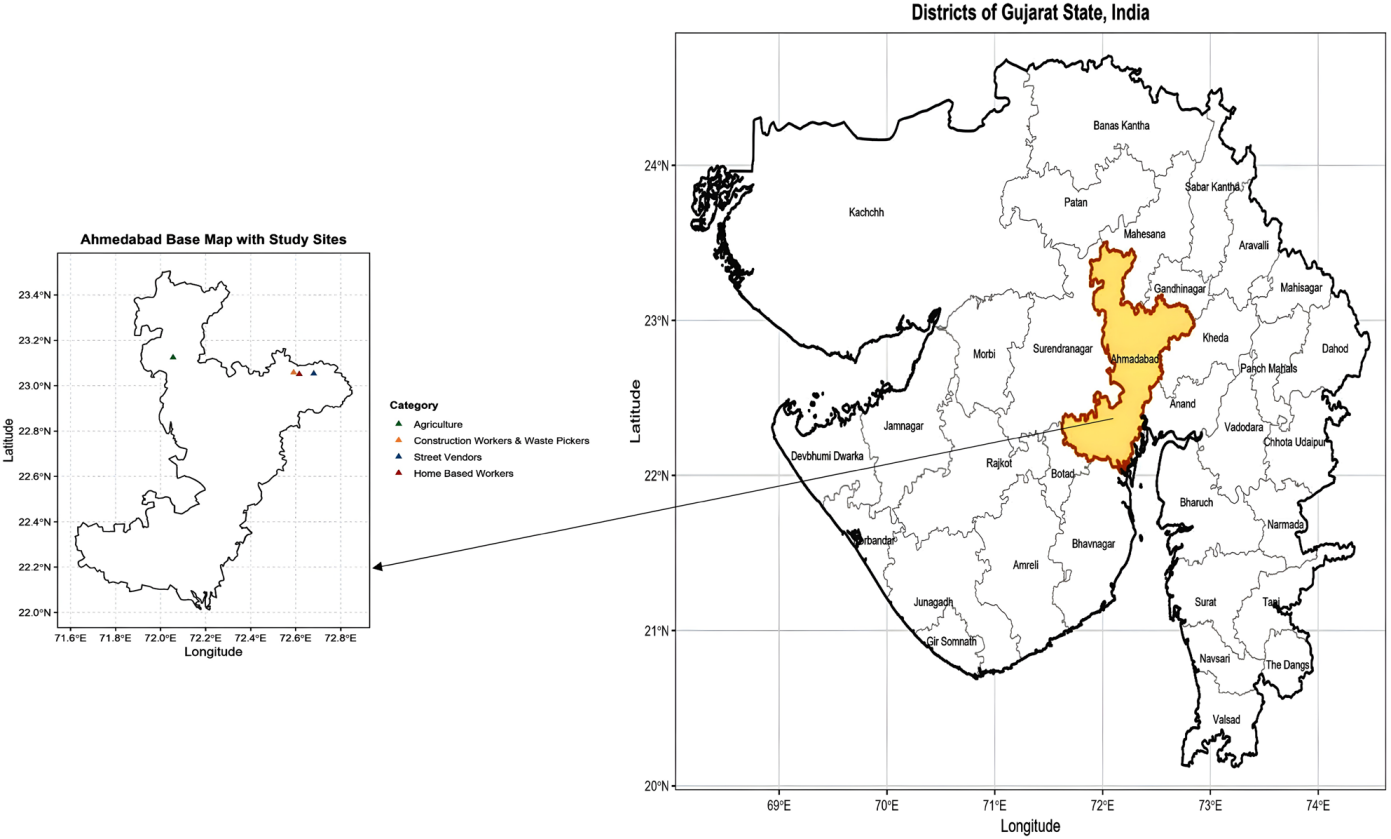
Study sites of focus group discussions with informal women workers in Ahmedabad district, Gujarat, India. The map illustrates the urban and rural locations within Ahmedabad district where focus group discussions were conducted across five occupational groups—agricultural workers (rural), and construction workers, street vendors, home-based workers, and waste recyclers (urban)

This qualitative study employed FGDs to explore the multi-level influences on health challenges and healthcare access among IWWs in Ahmedabad, Gujarat (India). Ahmedabad is one of India’s largest urbanising districts with a substantial informal workforce across diverse occupations. The district also presents substantial variability in urban and rural informal work settings, making it suitable for examining diverse socioecological influences on health. The study was conceptually guided by the SEM, examining intrapersonal, interpersonal, organizational, community, and policy-level determinants [12]. Five sites were purposively selected – four from urban areas [construction workers (CW), street vendors (SV), waste recyclers (WR), and home-based workers (HW)] and one from rural area [agricultural workers (AW)], based on the predominant work locations of each occupational group (Fig. 1). The study followed the Consolidated Criteria for Reporting Qualitative Research (COREQ) [13].

### Study participants

Participants were recruited from five occupational groups accounting for most informal women’s employment: AW, CW, SV, WR, and HW [2]. Among the selected groups, AW, CW, SV and WR primarily engaged in outdoor work, often under variable weather conditions and with limited access to shade or sanitation. AW were primarily involved in manual sowing, weeding, and harvesting activities in rural outskirts. CW engaged in physically demanding tasks such as carrying cement, mixing mortar, and bricklaying at construction sites. SV typically sold perishable goods like fruits, vegetables, and snacks in open markets or roadside locations. WR collected, sorted, and segregated household and industrial waste materials for resale. HW, in contrast, performed predominantly indoor tasks such as stitching, embroidery, beading, packaging, or other small-scale manual production activities carried out within their households.

Women aged 18 years and above who were currently employed in any of these occupation were eligible. Exclusions were serious illness, lack of fluency in Gujarati, or refusal of written consent. Accredited Social Health Activists (ASHAs) and Self-Employed Women’s Association (SEWA) coordinators assisted with community engagement and participant identification. Prior approval were taken from local authorities and community leaders.

### Sample size and composition

Five FGDs were conducted, one per occupational group, with 6–10 participants each (total *n* = 41). The group size was intentionally chosen to balance diversity of perspectives while maintaining an environment conducive to open discussion, allowing each participant sufficient opportunity to share experiences and ensuring active interaction among members. Discussions lasted 45–60 min in community venues ensuring privacy and comfort. Because each FGD represented a distinct occupational category, data collection focused on achieving within-group informational saturation. In all FGDs, no new ideas emerged in the later stages of discussion, indicating adequate depth within each group.

### Data collection tools and procedure

All FGDs were conducted in Gujarati, the primary language spoken by the participants. A semi-structured FGD guide was developed specifically for this study following a review of relevant literature and the domains of the Socioecological Model (SEM). The guide is provided in English as Supplementary File 1 to ensure transparency and replicability. Questions were framed under the five SEM levels [12]. The guide included questions on (i) **intrapersonal** (knowledge, attitudes, beliefs, personal experiences), and mental well-being expressed through locally relevant terms such as “tension” or “stress” or “constant worry”), (ii) **interpersonal** (family dynamics, social and peer support), (iii) **organizational** (workplace safety, sanitation, safety protocols), (iv) **community** (neighbourhood conditions, local norms, community organisations), and (v) **policy** (awareness and experiences with health and labour policies).

Potential participants were identified through collaboration with SEWA coordinators – a grassroots trade union representing women workers in the informal economy, ASHAs – community health workers under India’s National Health Mission, and local community leaders, who assisted in mapping occupational clusters and mobilizing eligible women workers. This participatory approach ensured trust-building, inclusivity, and representation of diverse informal occupations. FGDs were held in familiar and easily accessible community venues such as community halls, Anganwadi centres, or local panchayat offices. These neutral spaces provided a comfortable and safe setting that encouraged participation and candid discussions.

Each FGD was moderated by a trained qualitative research team comprising two female interviewers (ER, ZK) one male (AD), all fluent in Gujarati and experienced in community-based fieldwork. AD and ER hold Master’s in Public Health (MPH) degrees, while ZK is a trained research coordinator with extensive grassroots experience. Senior researchers AS (MD) and MT (MBBS) provided oversight on study design, quality assurance, and methodological guidance. While two female researchers facilitated the FGDs, the male team member (AD) primarily assisted with audio recording and logistical coordination. His role and presence were clearly explained to participants beforehand, and he did not lead or intervene in discussions. This arrangement ensured efficient facilitation and technical support without compromising participants’ comfort, openness, or confidentiality.

Written consent was obtained before audio-recording. Discussions encouraged open sharing in a respectful environment. Reflexive field notes were maintained by the research team immediately after each discussion to capture contextual nuances, group dynamics, and interviewer reflections. These notes were reviewed during analysis to identify and mitigate potential researcher bias and to enhance analytical depth. Reflexivity helped ensure transparency and reliability in interpreting the participants’ narratives [14]. 

### Data management and analysis

All FGDs were audio-recorded, transcribed verbatim in Gujarati, and subsequently translated into English by two researchers (AD and ER). Transcripts were reviewed for accuracy, completeness, and contextual fidelity before analysis.

We applied reflexive thematic analysis following Braun and Clarke’s six-step approach: familiarization with data, generation of initial codes, identification of themes, reviewing themes, defining and naming themes, and final synthesis [15]. The analysis was deductively informed by the SEM, which served as an organising framework for grouping codes and themes across its five levels. While SEM guided the structure of the analysis, theme development remained grounded in participants’ narratives, consistent with reflexive thematic analysis. Coding was carried out independently by two researchers (AD and ER) using Microsoft Excel. Codes were initially organized according to the five levels of the Socioecological Model (SEM) to ensure theoretical alignment.

Data collection continued until within-group informational saturation was achieved for each occupational category – that is, each FGD yielded no new insights or codes in the later stages of discussion. To enhance analytic rigor, coding discrepancies were discussed and resolved through consensus with senior researchers (MT, AS). This consensus-based approach ensured interpretive reliability and consistency in theme development. Final themes were reviewed and endorsed by all team members (AD, ER, MT, AS).

### Researcher credentials

The qualitative team comprised public health professionals and medical doctors with occupational health expertise. AD and ER (MPH) and ZK (field research coordinator) conducted FGDs and transcription; AS (MD) and MT (MBBS) provided senior oversight.

### Participant feedback on findings

Due to time and logistical constraints, transcripts and themes were not returned to participants for comment.

## Results

**Fig. 2:**
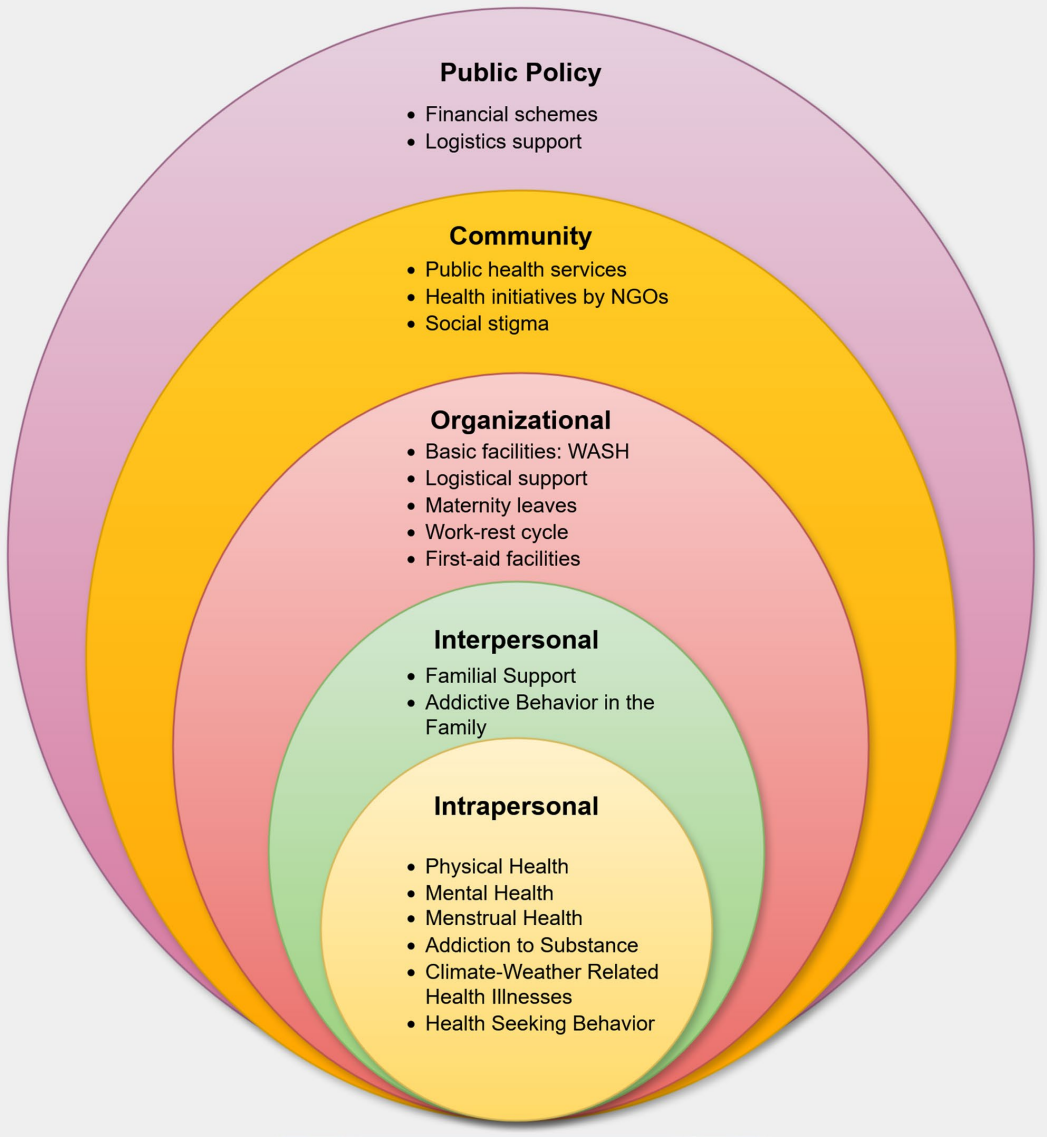
Conceptual framework illustrating the multi-level determinants of health among informal women workers based on the Socioecological Model (SEM). This socioecological model is intended as a conceptual framework to illustrate the multiple, interacting levels of influence on the health of informal women workers. While the diagram uses a concentric format for visual clarity, it does not imply a strict hierarchical or encapsulating relationship among the levels. In reality, these levels often interact dynamically and bidirectionally, with each exerting influence independently or in conjunction with others

A total of 41 women informal workers participated in five FGDs representing AW (*n* = 10), CW (*n* = 6), SV (*n* = 8), HW (*n* = 10), and WR (*n* = 7). Mean ages ranged from 35.4 to 49.6 years, with work experience from < 1 year to 27 years (Table 1). Except for AW, who were from rural areas, all other groups were urban-based. AW, CW, SV, and WR were primarily outdoor workers exposed to weather extremes, while HW mostly operated indoors or in semi-covered environments. Themes were mapped to the five domains of the SEM to illustrate the multi-level determinants influencing the health and wellbeing of informal women workers in Ahmedabad (Fig. 2; Table 2). Key findings are presented below, highlighting major thematic patterns within and across occupational groups. Thematic findings were broadly consistent across urban and rural groups, with no substantial variation in the nature of reported health challenges.

**Table 1:** Demographic details of the participants

Sector	No. of Participants	Age (years) Mean (SD)	Work Experience (Range in years)
Agricultural Workers *	10	38 (9.2)	1.7–27
Construction Workers	6	35 (11.1)	1–18.4
Street Vendors	8	49 (10.3)	5.1–17
Home-Based Workers	10	44 (7.5)	2.5–13
Waste recyclers	7	39 (7.9)	0.4–15

*All participants resided and engaged in work at urban locations, except for the agricultural workers, who were from the rural locations

**Table 2:** Summary of key themes across socioecological model (SEM) levels with representative participant quotes

SEM Level	Key Themes	Representative Quotes
Intrapersonal	Musculoskeletal pain, fatigue, menstrual and reproductive issues, stress and anxiety, heat-related illness, self-medication, and substance use as coping.	“Pulling heavy carts gives us leg, stomach, and back pain… My sister fainted once due to dizziness.” – SV 5 “It really pains a lot during periods… even if we want to take unpaid leave, we can’t afford it.” – WR 3
Interpersonal	Dual burden of work and household duties; limited family support; domestic conflict; alcohol use among male family members.	“There is so much pressure. We handle everything at home and then face deadlines at work.” – HW 3 “Sometimes, when our husbands come home after drinking alcohol, they fight with us.” – HW 9
Organizational (Workplace)	Lack of PPE, poor sanitation, absence of rest breaks, unsafe conditions, and no maternity protection.	“They don’t give us anything to wear on our hands while making cement products.” – CW 6 “We work even during pregnancy, up to the 8th month…maternity leaves are not a chance.” – CW 2
Community	Limited access to primary health care, preference for private clinics, inadequate infrastructure (toilets, water), social stigma, and role of NGOs.	“We avoid going to the PHC—it’s far and always crowded. We can’t afford to lose a day’s wage.” – SW 2 “Some people make fun of us… call us ‘kachara wala ben.’” – WR 1
Policy	Lack of social protection, need for pensions, financial aid, home-based work tools, and integration of OHS in primary care.	“We need pensions for when we can’t work anymore, and work-from-home options to support ourselves.” – CW 6 “If the government could provide sewing machines, it would help a lot.” – HW 4

### Intrapersonal factors

#### Physical health challenges

Across all groups, MSD from repetitive work, static postures, and heavy lifting were prevalent. AW and WR reported respiratory symptoms from dust and fumes; CW and AW noted skin damage from cement and chemicals—conditions normalized amid lacking protective equipment and training.

*Cement often causes cracks in my hands…it burns my hands a lot.* – *CW 6*

*We suffer from throat irritation and breathing difficulties due to dust*,* during castor harvesting.* – *AW 6*

*Pulling heavy carts gives us leg*,* stomach*,* and back pain. My sister fainted once had to be hospitalized.* – *SV 5*

#### Mental health and stress

Persistent anxiety, restlessness, and uncertainty were common themes. Participants described feeling “constantly worried” about meeting basic needs, managing debts, and balancing work with childcare and domestic duties. Anxiety about children’s education and financial insecurity to recover investments were prominent concerns. They felt emotional exhaustion described as “being drained” due to unrelenting pressure and responsibilities.

*We often experience tension/ stress. We invest a lot of money in crops*,* but there is always stress about whether we will even recover half of it. This uncertainty causes mental pressure. Physically too*,* agricultural activities lead to problems*,* but the financial worry is constant.* – *AW 7*

*There is a lot of pressure. The tension comes from household responsibilities – taking care of children*,* husband*,* cooking*,* and managing everything at home. On top of that*,* there is pressure to complete work tasks within deadlines. So yes*,* we feel restless and drained because of all these responsibilities combined*. – *HW 2*

*We earn barely enough to feed our families*,* yet we have to pay school fees. It’s stressful.* – *CW 4*

#### Menstrual and reproductive health

The women described experiencing menstrual pain but felt compelled to continue working because taking leave meant losing income which they could not afford every month. Some participants shared that even when leave was technically allowed, it was unpaid and therefore not a practical option. Poor sanitation worsened hygiene challenges.

*It really pains a lot during periods. If anyone has severe pain*,* she may take a day’s unpaid leave ….but we can’t afford it every month. Some of us think that taking leave actually increases mental stress*,* so it’s better to go to work.* – *WR 3*.

#### Substance use as a coping mechanism

Workers described relying on tobacco related products as a means to cope with the constant physical strain of their jobs. Substance use had gradually shifted from an occasional coping tool to a form of dependence. This pattern reflects how demanding work environments can normalize and reinforce addictive behaviours among workers.

*I’m addicted to chhikni powder. I enjoy using it while working*,* it gives me stimulation.* – *CW 2*

*It (substance) started bitter*,* but now it relieves my stress every time I take it*. – *WR 4*

#### Climate and weather-related illness

Outdoor workers described experiencing a range of heat-related symptoms during the summer, including dizziness, headaches, excessive thirst, and even episodes of nosebleeds. Many tried simple coping strategies such as seeking shade or covering their heads with sarees although these measures provided limited relief in the absence of proper shelter or resting spaces. Together, these accounts show how intense climatic conditions directly affect workers’ health and productivity, especially for those working outdoors.

*Last summer*,* I had a nosebleed from the heat. Hopefully*,* this year will be better.* – *SV 3*

*The heat creates many problems for us. We often sit under the shade whenever possible. We wear sarees to cover our heads to reduce the discomfort*,* but without shade and seating arrangements*,* it’s still difficult*. – *CW 6*

*It really gets worse to work mainly during the summer. We feel thirsty*,* dizzy. Some of us have blood pressure issues and we suffer from headaches as well.* – *WR 3*

#### Health-seeking behaviour

IWWs often choose private clinics over government health centers, perceiving their medicines as more effective. This preference, driven by trust and efficacy concerns, increases out‑of‑pocket costs, strains finances, and can delay or reduce use of public services, hindering integration of occupational health into primary care.

*We do take medicines from the sub-centre here in the village……however*,* we feel that government medicines take a longer time to show effect*,* whereas the medicines from private clinics provide instant relief.* – *AW 8*

### Interpersonal factors

#### Familial support and gender roles

Women described how limited emotional and social support within their households intensified both their physical and mental burden. Expected to manage all domestic responsibilities while simultaneously meeting work demands, they experienced constant pressure and exhaustion. All these experiences that reveal how entrenched gender norms and inadequate support systems systematically amplify women’s emotional distress and daily workload.

*There is so much pressure. We handle everything at home and then face deadlines at work. It’s overwhelming*. – *HW 3*

*We usually sat outside in the sunlight to reduce electricity bills. Sometimes*,* the bill increases suddenly….our family members scold us*,* saying we are wasting electricity. The sewing machine has a foot motor which does not work on mini light (low electricity).* – *HW 6*

#### Addictive behaviours in family members

Substance abuse among male family members contributed to household conflict, financial instability, and physical or emotional abuse. These dynamics not only affect their psychological well-being but also limit their ability to seek support, creating a cycle of vulnerability within the home environment.

*Sometimes*,* when our husbands come home after drinking alcohol*,* they fight with us* – *HW 9*

### Organizational factors

#### Lack of safety equipment and support

Workers reported inadequate or absent logistic support, including PPE and sewing machines. Most purchased their own equipment without external support.

*We have a sewing machine*,* we can do it*,* but we have other people who want to do it*,* but they don’t have a sewing machine. So*,* if they give us anything like that it would helpful*. – *HW 1*

*They don’t give us anything to wear on our hands. If we wear gloves while making cement products*,* then there won’t be a problem*.– *CW 6*

#### Absence of first aid

Injuries were typically managed informally using cloth or improvised remedies at the worksite, with formal medical care deliberately delayed by the workers until after completing their job tasks. This pattern reflects the economic pressures and absence of paid sick leave, where immediate care is deprioritized in favour of securing daily wages and avoiding income loss.

*When we get injured*,* we cover injured part with whatever cloth we have at that moment. After finishing our work*,* we go to the hospital.* – *CW 3*

#### Inadequate work-rest cycles

Participants reported that long working hours with minimal or no breaks resulted in persistent physical exhaustion. The combination of strenuous tasks, such as prolonged standing and repetitive manual labour, intensified bodily discomfort over the course of the day. Irregular work-rest cycle not only reduced their ability to recover but also increased their vulnerability to fatigue and work-related strain.

*If we have to stand for long hours*,* our legs hurt a lot. Even when climbing bricks*,* our whole body aches. The owner only gives us half an hour to sit during meals.……they don’t let us rest when we are tired.* – *CW 7*

#### Poor water, sanitation and hygiene (WASH) facilities

Women described open toileting and poor sanitation as major sources of distress, affecting not only their physical health but also safety and dignity. Many reported restricting fluid intake to avoid the need for urination during work, leading to dehydration and urinary problems. In some cases, lack of privacy during menstruation resulted in embarrassment and shame, reinforcing gendered vulnerabilities and unsafe coping strategies.

*During periods*,* when we need to use the washroom frequently and are far from the place*,* sometimes we have to go outside*,* behind trees*. – *WR 3*

*There are no toilets on farms. We just use open areas*. – *AW 4/5*

#### Lack of maternity leave

Women reported continuing strenuous work well into their late pregnancy because there was no provision for maternity leave or income support. This lack of protection placed pregnant workers in a vulnerable position, compromising maternal well-being.

*We work even during pregnancy*,* up to the 8th month. The employer will never pay a single penny when we are off from work*,* maternity leaves are not a chance. We never get maternity leaves*. – *CW 2*

### Community-level factors

#### Barriers to public healthcare access

Despite being eligible for services at urban health centres (UHCs), and awareness of the Pradhan Mantri Jan Arogya Yojana (PMJAY)—India’s government-funded health insurance scheme, women often chose private clinics due to the long distances, long wait times, and perceived inefficiency of public services. For daily-wage earners, long waiting periods often translate into wage loss, further disincentivizing public healthcare services uptake.

*Some of us have the Ayushman Bharat (PMJAY) card*,* while a few others do not have this card*. – *SV 3*

*We avoid going to the UHC—it’s far and always crowded. We can’t afford to lose a day’s wage waiting.* – *SW 2*

#### Role of NGOs and local initiatives

Participants acknowledged support from local organizations like SEWA for health awareness and seasonal relief. Such local initiatives were viewed as essential stopgaps in the absence of formal institutional services.

*SEWA gave us ORS and health tips in the summer. Nothing came from the government*. – *CW 4*

#### Social stigma and discrimination

Workers, particularly WR reported feeling stigmatized and stereotyped. These not only undermined their dignity but also contributed to emotional distress and feelings of social exclusion, thereby intersecting with mental health challenges reported by the participants.

*Some people make fun of us….Oye kachara wala ben (In English: Hey*,* garbage collector) come here*,* pick this up*,* collect it*,* take this. Some see us as if we have come to steal something. ‘Why did you come here?’*,* ‘Did you come here to steal?’*,* ‘Why did you take this?’…If anything disappears then they just blame us that you took this thing from here.* – *WR 1*

### Public policy-level factors

#### Demand for financial and social protection

Two distinct demands were identified across worker groups. Outdoor, contract-based workers such as those in construction expressed concerns that as they aged, employers would no longer recruit them for physically strenuous tasks, emphasizing the need for financial security through pensions or opportunities to transition into home-based income-generating activities. Conversely, HW sought indirect financial support in the form of tools and equipment – particularly sewing machines – to sustain or expand their home-based enterprises and reduce dependency on intermediaries for work opportunities.

*We need pensions for when we can’t work anymore*,* and work-from-home options to support ourselves*. – *CW 6*

*Many women want to do home-based work but don’t have machines. If the government could provide them*,* it would help a lot*. – *HW 4*

## Discussion

This qualitative study employed the multi-level SEM to examine the multifaceted health challenges faced by IWWs across five major occupational groups—AW, CW, SV, HW, and WR—in Ahmedabad, India. The findings demonstrate a convergence of occupational, environmental, structural, and gender-based vulnerabilities that together shape the physical, mental, and social well-being of these women.

MSDs emerged as a predominant health concern, commonly linked to poor ergonomics, prolonged static postures, and repetitive tasks [3, 16]. These findings are supported by a systematic review that reported a higher prevalence of MSDs among IWWs [17]. HW, in particular, described headaches and dizziness from sustained visual and manual strain [3]. Exposure to pesticides in agriculture and cement dust in construction was linked to respiratory conditions, skin irritation, and cracked skin [18]. The hazards of such work intensifies in the absence of personal protective equipment (PPE) [19]. Some participants reported unconventional coping strategies, such as applying nail polish to soothe cement-induced skin damage, underscoring both poor health literacy and limited access to occupational health services.

Mental health challenges were initially underreported, but upon probing, participants disclosed persistent anxiety, restlessness, and emotional exhaustion—driven by financial insecurity, job instability, caregiving responsibilities, and unsafe workplaces. These findings align with prior studies linking informal employment to increased risk of poor mental health [20]. Wage structures varied across occupations, with HW paid per piece, and CW and AW on daily wages. SV and WR relied on uncertain earnings from sales. This economic vulnerability often forced women to continue working despite health issues, further compounding physical and psychological strain [18, 21]. 

Climate and environmental exposures significantly influenced occupational health, especially for outdoor workers. Heat-related illnesses such as dehydration, dizziness, and heat stroke were common in summer, while respiratory symptoms and injuries increased during winter and monsoon conditions. SV reported reducing fluid intake to avoid using non-existent toilet facilities, heightening the risk of dehydration and heat stroke [22, 23]. Moreover, urban informal workers also faced water scarcity and poor sanitation [24]. Evidence indicates that each 1 °C rise in temperature can increase sickness probability by 5–7% and medical costs by 14% [25]. Climate-related health risks were closely intertwined with socioeconomic disadvantage and inadequate urban infrastructure.

Healthcare access was marked by mixed use of public and private services. Although most participants were aware of public health schemes, a clear preference emerged for private clinics, perceived to offer higher-quality and faster-acting medicines despite higher out-of-pocket costs. Public facilities were considered inefficient and time-consuming, with long wait times leading to income loss for daily-wage workers. Similar patterns have been noted in Ghana and Delhi, where informal workers opt for private care due to concerns about quality and timeliness in public services [26, 27]. 

Awareness of the Pradhan Mantri Jan Arogya Yojana (PMJAY) – part of the Ayushman Bharat program – was high, but utilization was limited. PMJAY is India’s government-funded health insurance scheme [28]. For serious treatments, participants relied more on government-subsidized health entitlement cards to reduce inpatient costs, while routine and minor ailments were managed in private facilities. Lack of preventive and outpatient coverage under many government schemes was a notable gap, given these represent a large share of healthcare costs for IWWs. Community health workers and NGOs were regarded as valuable for health education and linking workers to available services.

This study also revealed widespread socio-economic and workplace barriers similar to report from earlier studies such as low and irregular wages [29], long working hours, no paid leave and minimal social protection [30]. Many were sole earners, making withdrawal from work practically impossible. Workplace sanitation was frequently inadequate, with no toilets or clean drinking water. Some women restricted water intake to avoid needing toilets. These findings align with prior evidence on infrastructure gaps severely affecting IWWs [31, 32]. Underpayment, lack of formal employment contracts, and high production pressure were widespread [25]. Maternity protection was absent; participants reported working into the third trimester without accommodations or financial security [5]. 

Safety and immediate care provisions were minimal. First aid facilities were rarely available; instead, women relied on self-medication or home remedies, risking complications from untreated conditions. These practices reflected both low health literacy and weak integration with formal health systems.

Worker recommendations reflected a desire for both economic and occupational health interventions. Home-based income-generating opportunities—especially for older women—were suggested to reduce physical strain while sustaining livelihoods. Requests for pension schemes, financial security programs, and logistical support (including sewing machines and PPE) were common. Barriers to accessing government welfare benefits included limited literacy, migratory status, and lack of identity documentation.

Although India has enacted welfare policies such as the Unorganized Workers’ Social Security Act (2008) [33] and launched platforms like e-Shram to register informal workers [34], our findings indicate limited awareness and uptake of these schemes. Similarly, while Pradhan Mantri Shram Yogi Maan Dhan Yojana [35] offers pension support, Ayushman Bharat provides health insurance [28], and Pradhan Mantri Matru Vandana Yojana offers maternity incentives [36], these programs are underutilized by the target study group due to awareness gaps, administrative barriers, and poor alignment with the lived realities of IWWs.

The study underscores how gendered social roles intersect with occupational risks to shape women’s health vulnerabilities. Unlike their male counterparts, women workers in informal employment shoulder dual burdens of paid and unpaid labour, face mobility restrictions, and often experience wage discrimination. These gendered dynamics exacerbate fatigue, stress, and exposure to unsafe working conditions. Our findings align with prior studies emphasizing the need for gender-responsive occupational health policies that recognize women’s multiple roles and structural disadvantages within informal labour systems [7]. 

Findings highlight the need to integrate occupational health within India’s primary health care framework by (i) incorporating occupation-related screening questions into routine check-ups, (ii) building capacity of community health workers and staff to identify and manage occupation-related illnesses, and (iii) creating referral linkages with specialized occupational health centres. Embedding such mechanisms within primary health care services can bridge health system gaps and extend equitable care to informal women workers.

### Strength and limitations

A key strength of this study lies in its use of the Socioecological Model (SEM) to systematically explore the multi-level determinants of health among IWWs across five major occupational groups in Ahmedabad, yielding occupationally nuanced and gender-sensitive insights. Focus group discussions in the local language enabled rich, context-specific narratives from a population that is often underrepresented in health systems research.

Limitations include its focus on a single district, which may not reflect regional diversity, and potential underreporting of sensitive issues like mental or reproductive health due to stigma. As a qualitative study, findings are not generalizable but provide transferable evidence to guide localized, gender-responsive health policies and interventions.

### Policy translation and engagement

As an immediate step toward policy translation, the findings were presented at a national roundtable discussion convened in collaboration with key stakeholders, including representatives from the Ministry of Labour and Employment (MoLE), National Health Systems Resource Centre (NHSRC), International Labour Organization (ILO), Central Board of Welfare Education for Workers (CBWE), V. V. Giri National Labour Institute (VVGNLI), ICMR-NIOH, Lok Swasthya SEWA Trust (LSST-SEWA), Women in Informal Employment: Globalizing and Organizing (WIEGO, UK), Worker Unions and workers.

Discussions, grounded in the lived experiences of IWWs, produced priorities including integrating occupational health into Ayushman Bharat and primary care, expanding social protection, addressing climate-related heat impacts (especially on women’s health), and building primary healthcare capacity in occupational risk assessment. Participants also urged inclusion of informal sector OHS modules in medical and nursing curricula, highlighting the value of participatory, community-driven research in shaping health and labour policies for vulnerable workers (Box 1).

**Box 1:** Recommendations for strengthening occupational health systems for informal women workers

- Integrate screening for common work-related illnesses among informal women workers into existing primary healthcare services, especially at Health and Wellness Centres (HWCs) under Ayushman Bharat scheme. - Build capacity of primary care providers to assess occupational risks and deliver gender-sensitive counselling and preventive care. - Strengthen social protection linkages to ensure access to welfare and insurance benefits.
- Improve workplace infrastructure such as sanitation, drinking water, and shade to reduce heat and ergonomic stress. - Promote inclusion of occupational health modules in medical, nursing, and public health curricula with focus on informal sector challenges.

## Conclusion

Women in India’s informal economy face intertwined occupational, environmental, and structural health risks that remain largely invisible to existing health systems. This study underscores the urgent need to integrate occupational health within India’s primary healthcare framework through gender-responsive and context-specific approaches. Strengthening the capacity of frontline health workers to identify, screen, and manage work-related illnesses can bridge critical service gaps. Beyond service delivery, structural reforms are essential to address the social determinants of health for these workers. Expanding social protection coverage, ensuring maternity and wage security, and improving workplace infrastructure for water, sanitation, and safety are necessary to reduce vulnerability. All these measures can promote long-term sustainability to protect the country’s most invisible yet indispensable workforce.

## Supplementary Information

Below is the link to the electronic supplementary material.

Supplementary Material 1

## Data Availability

The data that support the findings of this study are available on reasonable request from the corresponding author.
