# Supplementary material for: Toward inclusive primary health care: understanding health needs of women in India’s informal economy through a socioecological framework
Source: BMC Health Serv Res. 2025 Dec 6;26:53. doi: 10.1186/s12913-025-13855-7 (PMC12797478; doi:10.1186/s12913-025-13855-7)
Supplement: Supplementary file 1 — Supplementary Material 1 [file 12913_2025_13855_MOESM1_ESM.pdf]

Thematic Guide

Start with introducing yourself, ask everyone their names, about their family members – nuclear/joint, the youngest – oldest person at home, about their native place, currently residential place, education, current occupation, financial condition, persons working in the family, to build rapport and later move forward to the following questions.

SEM Domain	Questions	Prompts
**Intrapersonal**	What health problems have you experienced as a result of your work?	Any kind of physical health issues due to work (Body pain, fatigue, respiratory problems, skin issues, menstrual problems, Musculoskeletal Disorders, etc.)
	Have you or your co-workers faced accidents or injuries?	Nature of injuries, reporting system, responses
	How does your work affect your mental and emotional well-being?	Familial-social tension or stress related to financial crisis (Stress, anxiety, depression, workplace conflict, job insecurity)
	What would you feel about the weather condition nowadays?	Heat Wave/Cold waves/Heavy rain, etc.
	What do you know about the health risks associated with your work?	Sources of information, personal understanding of risk
	How do you manage your physical and mental health issues?	Do you go to hospital for the treatment (Traditional remedies, rest, coping mechanisms, etc.), are you addicted to any substance?
**Interpersonal**	What kind of support do you receive from your family or friends regarding your health or work-related stress?	Emotional support, household help, financial support, substance abuse
	How do your relationships at work (with peers/employers) affect your well-being?	(Physical/verbal harassment, cooperation, communication)
**Organizational**	What kind of safety measures or protective equipment are available at your workplace?	Gloves, masks, training, shaded areas, breaks

	Can you tell me about your daily work routine, including how often you take breaks during your working hours?	Total working hours, regular breaks for rest, meals, or to drink water. Who decides when and how long you can rest?
	Can you describe the water, sanitation, and hygiene facilities available at your workplace?	Clean and safe toilets to use - separate for men and women? Clean drinking water, soap and water to wash hands?
	What kind of first aid support or medical help is available at your workplace in case of an injury or health emergency?	Who is responsible to make it available? Has anyone ever required it? What happened then?
	Are there any health check-ups or education sessions provided by your employer or workplace?	Awareness programs, preventive services
**Community**	Can you describe any challenges you face when trying to access public healthcare services?	Difficulties at government hospitals or clinics, and issues related to distance, cost, documents, or staff behaviour, etc.
	Have you received any support from local NGOs or community groups for health issues?	Awareness, referrals, material aid
	Can you please describe about the society and your community behaviour with you and your family?	Societal stigma due to occupation,
**Policy**	Which government schemes or policies that support informal workers' health are you aware of?	Insurance schemes, maternity benefits, labour laws
	Have you received any benefits or entitlements from government programs?	Ease of access, coverage, limitations
	Is there anything you'd like to suggest to improve your health experiences or other kind of support?	Reflections on challenges, demand and suggestions for change
